# Biomarkers beyond BRCA: promising combinatorial treatment strategies in overcoming resistance to PARP inhibitors

**DOI:** 10.1186/s12929-022-00870-7

**Published:** 2022-10-25

**Authors:** Yu-Yi Chu, Clinton Yam, Hirohito Yamaguchi, Mien-Chie Hung

**Affiliations:** 1 Department of Molecular and Cellular Oncology, The University of Texas MD Anderson Cancer Center, Houston, TX 77030 USA; 2 Department of Breast Medical Oncology, The University of Texas MD Anderson Cancer Center, Houston, TX 77030 USA; 3 Department of Translational Molecular Pathology, The University of Texas MD Anderson Cancer Center, Houston, TX 77030 USA; 4 Research Center for Cancer Biology, and Center for Molecular Medicine, Graduate Institute of Biomedical Sciences, China Medical University, 100, Sec 1, Jingmao Rd., Beitun, Taichung, 40402 Taiwan, ROC; 5 Department of Biotechnology, Asia University, Taichung, 413 Taiwan

**Keywords:** PARP inhibitor, Resistance to PARP inhibitor, Biomarkers, PARPi-based combination strategies, Marker-guided effective therapy (Mget)

## Abstract

Poly (ADP-ribose) polymerase (PARP) inhibitors (PARPi) exploit the concept of synthetic lethality and offer great promise in the treatment of tumors with deficiencies in homologous recombination (HR) repair. PARPi exert antitumor activity by blocking Poly(ADP-ribosyl)ation (PARylation) and trapping PARP1 on damaged DNA. To date, the U.S. Food and Drug Administration (FDA) has approved four PARPi for the treatment of several cancer types including ovarian, breast, pancreatic and prostate cancer. Although patients with HR-deficient tumors benefit from PARPi, majority of tumors ultimately develop acquired resistance to PARPi. Furthermore, even though *BRCA1/2* mutations are commonly used as markers of PARPi sensitivity in current clinical practice, not all patients with *BRCA1/2* mutations have PARPi-sensitive disease. Thus, there is an urgent need to elucidate the molecular mechanisms of PARPi resistance to support the development of rational effective treatment strategies aimed at overcoming resistance to PARPi, as well as reliable biomarkers to accurately identify patients who will most likely benefit from treatment with PARPi, either as monotherapy or in combination with other agents, so called marker-guided effective therapy (Mget). In this review, we summarize the molecular mechanisms driving the efficacy of and resistance to PARPi as well as emerging therapeutic strategies to overcome PARPi resistance. We also highlight the identification of potential markers to predict PARPi resistance and guide promising PARPi-based combination strategies.

## Background

The Poly (ADP-ribose) polymerase (PARP) family is essential for regulation of many critical cellular processes, including DNA damage response, transcription, chromatin remodeling, metabolism and inflammation [1–3]. To date, 17 members have been identified in the PARP family based on their homology to PARP1, the most well-characterized PARP protein which is responsible for more than 80% of Poly(ADP-ribosyl) (PAR) activity in the cell [4]. The well-known function of PARP1 is to initiate DNA repair by inducing PARylation, one of the post-translational modifications, in other proteins and itself [5]. PARP1 contains three functional domains including the DNA-binding domain, automodification domain and catalytic domain. The first two Zinc fingers in DNA-binding domain are critical for the binding of PARP1 on DNA damage sites and the third Zinc finger plays a key role in alteration of DNA-dependent PARP1 enzyme activity [6–8]. The glutamate and lysine residues in the central automodification domain are the acceptor sites for PARP1 to PARylate itself [9, 10]. Importantly, the catalytic domain in C-terminal of PARP1 is responsible for the transfer of ADP-ribose subunits from nicotinamide adenine dinucleotide (NAD^+^) to protein substrates and building up the negatively charged PAR chains [11]. The PARP1-mediated PARylation serve as a platform for recruiting the downstream repair proteins for repair DNA breaks [12]. Additionally, auto-PARylation of PARP1 is a critical step for successfully completing DNA repair and preventing the replication fork collapse caused by PARP1 trapped on damaged DNA [12]. In 2000s, the scientific focus on PARP1 transitioned from validating its molecular functions to identifying its physiological and pathological role in human cancer [13]. In addition, significantly increased expression of PARP1 and PARylation have been detected in malignant tumors of various cancer types [14, 15]. Based on these findings, PARP1 became the attractive therapeutic target for the treatment of cancer. Of note, remarkable studies in 2005 demonstrated that PARP inhibition selectively kills the *BRCA1/2* mutant tumor cells [16, 17], leading to the rapid clinical development of PARP inhibitors (PARPi) for patients with homologous recombination (HR)-deficient cancer. PARPi are the nicotinamide analogs which compete with NAD^+^ for the catalytic binding sites on PARP molecules to inhibit the PARylatioin and induce PARP trapping activity [18]. Currently, there are four PARPi approved by the U.S. Food and Drug Administration (FDA) for the treatment of different types of cancer. Although these PARPi have promising clinical activity through prolonging the survival of a board population of cancer patients, resistance to PARPi remains a significant clinical challenge. Therefore, a better understanding of mechanisms of resistance to PARPi and identification of reliable biomarkers to predict PARPi resistance are necessary for the development of marker-guided effective therapy (Mget) to overcome PARPi resistance.

## BRCA1/2 mutation and defective homologous recombination repair in cancer

Defects in many of genes encoding DNA repair proteins are commonly identified in human cancer [19]. Compromised DNA damage repair pathways in cancer cells result in genomic instability and leads to cancer development [20]. Of note, two tumor suppressor genes with critical roles in double-strand break (DSB) repair, *BRCA1* and *BRCA2*, are frequently mutated or deleted in several cancer types [21]. In 1990, a geneticist, Mary-Claire King discovered the *BRCA1* gene locus and its linkage to hereditary breast and ovarian cancer [22]. In 1994, the *BRCA1* gene was cloned [23], and in the same year *BRCA2* was also identified [24]. Till now, numerous studies have demonstrated that mutations of *BRCA1/2* increase the lifetime risk of breast or ovarian cancer development. Specifically, a healthy woman who harbor germline mutations of *BRCA1/2* have a 60–70% increased risk of breast cancer development and a 15–40% increased risk to develop ovarian cancer [25, 26]. Mutations of *BRCA1/2* genes have also been found in many sporadic tumors including pancreatic [27, 28] and prostate cancer [29, 30]. In addition to *BRCA1/2* aberrations, other genes involved in HR repair such as *RAD51C*, *RAD51D*, *PALB2* and *BRIP1,* are known to be mutated in many cancer types [31]. HR repair is a critical process for repairing the most cytotoxic DNA lesions, DSBs. When DSBs occur, ATM kinase is activated by the MRN complex (MRE11, RAD50 and NBN) and then phosphorylates the down-stream effectors including BRCA1 to promote the HR activity [32–35]. BRCA1 is a key regulator required for generating single-strand DNA (ssDNA) and recruiting the PALB2-BRCA2 complex. With the help of the PALB2-BRCA2 complex at DNA repair sites, replication protein A (RPA) is displaced and then replaced by the RAD51 recombinase [36]. The assembly of RAD51 filaments promotes homology sequence searching and base pairing to accurate repair [37]. Dysfunction of HR repair resulting from these HR gene mutations leads to less effective, error-prone non-homologous end joining (NHEJ) repair and gives rise to severe chromosomal instability that is associated with tumor development. Moreover, alterations of essential HR repair factors can result in phenotypic features similar to those caused by *BRCA1/2* mutations, giving rise to the term “BRCAness” [38]. Although the defects of DSB repair machinery due to BRCAness phenotype is associated with a higher risk of developing breast and ovarian cancer, patients with these tumors benefit from therapeutic strategies aimed at targeting the compromised DNA repair pathways to kill tumor cells through the accumulation of unrepaired DNA damage.

## Synthetic lethality between HR-deficiency and PARP inhibition

HR-deficient tumors have been found to be highly sensitive to DNA damage drugs such as platinum-based chemotherapy, which is frequently used as a part of the standard of care for patients with ovarian cancer. Two landmark studies published in 2005 [16, 17], first described the synthetic lethal interaction between *BRCA1/2*-deficiency and inhibition of PARP, offering a promising new approach over conventional chemotherapy for patients with HR-deficient tumors. PARP1 is a nuclear protein regulating base excision repair (BER) through PARylation [1]. Following DNA damage and sense of ssDNA breaks, PARP1 binds to the DNA damage sites and induces PARylation events to recruit multiple downstream DNA repair factors [12]. During this recruiting process, PARP1 auto-PARylates itself for releasing DNA-bounded PARP1 and allowing the DNA repair proteins to access and complete DNA repair [12]. Thus, inhibition of PARP1 results in the accumulation of unrepaired ssDNA break and replication fork collapse, which subsequently induce DSBs during DNA replication [17]. The persistence of DSBs is normally repaired by HR repair in the S phase of cell cycle [39]. BRCA1 and BRCA2 are the essential factors in regulating the HR repair pathway which is the largely error-free repair of DSBs [40]. For tumor cells with *BRCA1/2*- or HR- deficiency, PARP1 activity is important for preventing the spontaneous ssDNA breaks that results in accumulation of DSBs. Therefore, pharmacological inhibition of PARP selectively kills HR-deficient tumor cells by inducing the genomic instability and cell cycle arrest, ultimately leads to the synthetic lethality between PARP inhibition and HR deficiency. Together, these findings provide mechanistic insight and rationale for targeting compensatory DNA repair pathways as therapeutic strategies in cancer.

## FDA approval of PARP inhibitors

Currently, four small-molecular PARPi have been approved by FDA for tumors with *BRCA1/2* mutation or HR deficiency, including olaparib, rucaparib, talazoparib and niraparib (Table 1). Olaparib was the first PARP inhibitor approved for patients with *BRCA1/2* mutant, advanced-stage ovarian cancers in 2014. Of note, retrospective analysis of results from a clinical trial demonstrated that olaparib also improve progression-free survival (PFS) in the patients with *BRCA1/2*-wild type ovarian cancer [41]. These data suggest that the expanded biomarkers are needed for identifying patients with *BRCA1/2*-wild type tumors who might benefit from PARPi maintenance therapy. PARPi maintenance therapy is the ongoing treatment of tumors with PARP inhibitor after tumor has responded to the first-line treatments of chemotherapies. In 2017 to 2018, niraparib [42], olaparib [43] and rucaparib [44] have been approved for maintenance therapy in patients with platinum-sensitive ovarian cancer due to the observations that PARPi maintenance therapy significantly improves the PFS of patients with ovarian cancer regardless of *BRCA1/2* status. On the basis of above findings, some of tumors with wild type-*BRCA1/2* might contain deficiency of other genes involved in the HR repair pathway. Indeed, niraparib was approved for patients with HR deficient-ovarian cancer in 2019 [45]. In addition to ovarian cancer, the use of PARPi was also extended to other cancer types including breast, pancreatic and prostate cancer. In 2018, olaparib became the first PARP inhibitor to be approved for patients with HER2-negative, germline *BRCA1/2*-mutated, metastatic breast cancer [46]. Following the approval of olaparib in breast cancer, the FDA also approved another PARP inhibitor, talazoparib for patients with germline *BRCA*-mutated, HER2‑negative locally advanced or metastatic breast cancer in the same year [47]. Most recently, olaparib was further approved for adjuvant treatment of patients with *BRCA1/2*-mutated, high-risk HER2-negative early breast cancer [48]. In 2019, olaparib was also received FDA approval for the maintenance therapy in patients with *BRCA1/2* mutated pancreatic cancer [49]. Subsequently, olaparib and rucaparib were approved for patients with metastatic castration-resistant prostate cancer that is deficient in HR repair in 2020 [50, 51].

**Table 1:** FDA approvals of PARP inhibitors in cancer therapies

Name	Manufacturer	FDA approvals		Trial
Olaparib (Lynparza)	AstraZeneca	Ovarian	2014—Olaparib capsules in patients with BRCA1/2 mutant advanced-stage ovarian cancers who have received $\ge$ 3 types of chemotherapies	Phase II trial study (Kaufman et al. 2015)
			2017—Maintenance therapy for advanced -ovarian cancer patients with PR or CR to platinum-based chemotherapy	SOLO2/ENGOT-Ov21 (NCT01874353)
			2018—First line maintenance therapy for patients with BRCA1/2 mutant advanced-stage ovarian cancers	SOLO-1 (NCT01844986)
		Breast	2018—Patients with BRCA1/2 mutant HER2-negative metastatic breast cancer who have been treated with chemotherapy	OlympiAD (NCT02000622)
			2022—Patients with BRCA1/2 mutant HER2-negative high-risk early breast cancer who have been treated with adjuvant chemotherapy	OlympiA (NCT02032823)
		Pancreatic	2019—Adult patients with germline BRCA-mutated metastatic pancreatic adenocarcinoma	POLO (NCT02184195)
		Prostate	2020—Adult patients with HRR gene mutated metastatic castration-resistant prostate cancer	PROfound (NCT02987543)
Rucaparib (Rubraca)	Clovis Oncology	Ovarian	2016—Patients with BRCA1/2-mutant ovarian cancer refractory to ≥ prior lines of treatment	ARIEL2 (NCT018191344)
			2018—Maintenance treatment of patients with recurrent ovarian cancer	ARIEL3 (NCT01968213)
		Prostate	2020—BRCA-mutated metastatic castration-resistant prostate cancer	TRITON2 (NCT02952534)
Niraparib	Tesaro	Ovarian	2019—Patients with HR deficiency -positive, advanced ovarian cancer	QUADRA (NCT02354586)
			2020—First-line maintenance treatment of patients with advanced ovarian cancer	PRIMA (NCT02655016)
Talazoparib	Pfizer	Breast	2018—Patients with germline BRCA-mutated, Her2-negative locally advanced or metastatic breast cancer	EMBRACA (NCT01945775)

**Fig. 1:**
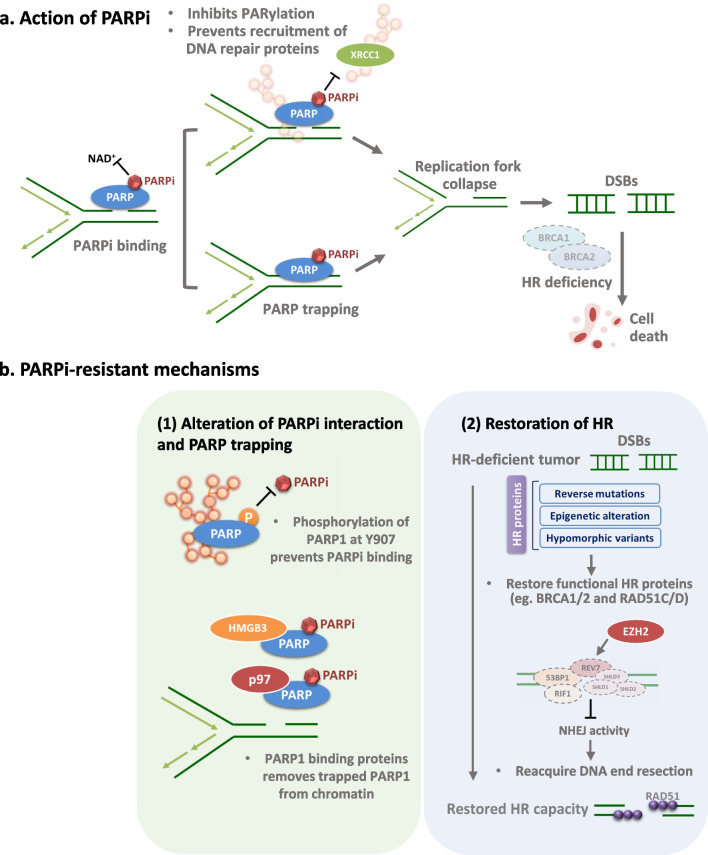
The molecular mechanisms driving the efficacy of and resistance to PARPi. **a** PARPi compete with NAD^+^ to inhibit PARylation of PARP target proteins and induce PARP trapping on damaged DNA, which in turn results in replication fork collapse and accumulation of toxic DSBs in HR-deficient cells. **b** General PARPi-resistant mechanisms. (1) PARPi resistance caused by phosphorylation and binding partners of PARP1. (2) Restoration of HR capacity occurs via re-expressing functional HR repair proteins (upper panel) or acquiring DNA end resection (lower panel)

The efficacy of PARPi is associated with their binding activity to the NAD^+^ binding site of PARP1 and ability to induce trapping of PARP1 on DNA that impair BER activity and induce the stalled replication fork, respectively [18]. The mechanism of action of PARPi was originally attributed largely to catalytic inhibition of PARP1 activity, reducing PARylation and blocking the recruitment of repair proteins, such as XRCC1 and DNA ligase III, which eventually impair the single-strand break repair (Fig. 1a). In recent years, more and more studies demonstrate that PARP trapping is critical for the anti-tumor activities of PARPi. The key role of PARP trapping in PARP inhibitor-mediated anti-tumor activity is consistent with the observation that treatment with PARPi results in greater cytotoxicity compared with PARP1 depletion alone [52]. One of the working mechanisms of PARPi mediated PARP trapping is that PARPi competitively bind to NAD^+^ binding pocket on PARP molecules to inhibit the auto-PARylation of PARP and prevent the dissociation of PARP from DNA [53]. The trapped PARP on DNA damage sites results in replication fork collapse and subsequently leads to the formation of DSBs (Fig. 1a). In addition, a recent study further discovered the molecules which are important for cytotoxicity caused by PARPi-mediated PARP trapping by utilizing the CRISPR-based screening approach [54]. Mechanistically, the genome-embedded ribonucleotides serve as a source of DNA lesions for PARP trapping, which are removed by RNaseH2 through the ribonucleotide excision repair pathway [54]. Since RNaseH2 was found to remove the genome-embedded ribonucleotides, this study further determined the frequency of RNASEH2B deletions in cancer patients. Importantly, *RNASEH2B* deletions were present in 43% of chronic lymphocytic leukemia (CLL) and 34% of castration-resistant prostate cancers (CRPCs) samples, suggesting these tumors have higher frequency of genome-embedded ribonucleotides and hypersensitivity to PARP inhibitors [54]. Therefore, RNase H2 protects cells from such DNA lesions, while loss of RNaseH2 induces an alternative pathway mediated by the topoisomerase 1 that cleavages misincorporated nucleotides, thereby causing DNA lesions on which PARP is trapped after PARPi treatment [54].

While these four FDA-approved PARP inhibitors have been widely used for treating tumor with HR deficiency, these first-generation PARP inhibitors are associated with hematologic toxicities due to inhibition of PARP2. Of note, a previous study demonstrated that loss of PARP2 but not PARP1, results in chronic anemia, highlighting the importance of developing selective PARP1 inhibitors [55] such as AZD5305 which has demonstrated a wide therapeutic index and limited toxicity in early clinical trials [56]. AZD5305 is known to exert anti-tumor efficacy by inhibition of PARylation, PARP trapping and growth inhibition. Importantly, AZD5305 selectively kills tumor cells with HR deficiency and exhibits limited cytotoxicity in normal cells [57]. Compared with first-generation FDA-approved PARPi, AZD5305 demonstrates better efficacy, greater target inhibition and improved tolerability [56].

## Resistant mechanisms of PARPi

Although PARPi have been demonstrated to have the promising clinical activity in patients harboring HR-deficient tumors, resistance to PARPi remains a significant clinical challenge. PARPi resistance has been found to arise from inhibition of PARPi-PARP interaction and PARP1 trapping activity (Fig. 1b, left panel). Notably, a receptor tyrosine kinase (RTK), c-MET was demonstrated to directly interact with PARP1 and phosphorylate it at tyrosine 907 (Y907), which induces PARylation of PARP1 and decreases the binding activity of PARPi, thereby rendering tumors resistant to PARPi [58]. A study further provides the clinical evidence to link the PARPi resistance and cytotoxic trapped PARP [59]. The PARP1 p.R591C mutation that inhibits PARP1 trapping ability was identified in a patient with ovarian tumor resistant to olaparib [59]. Moreover, recent studies found that PARP1 associated proteins such as the ubiquitin-dependent ATPase, p97, [60] and HMGB3 [61] facilitate the removal of trapped PARP1 from chromatin. Inhibition of these two PARP1-binding partners prolongs PARP1 trapping and sensitized cancer cells to PARPi [60, 61]. A selective and orally bioavailable inhibitor of p97, CB-5083 led to marked increase of talazoparib sensitivity in a patient-derived tumor organoid model derived from a patient with BRCA1 mutated TNBC, suggesting the potential therapeutic effect of combined treatment of p97 and PARP inhibitors in cancer patients [60].

Restoration of HR activity in HR-deficient tumor cells is the most common mechanism of acquiring resistance to PARPi. Reactivation of HR through secondary mutations or epigenetic regulation of *BRCA1/2* is frequently identified and has been found in patients with ovarian [62–65], breast [63, 64, 66], pancreatic [67] and prostate [68, 69] cancer with PARPi-resistant disease. The secondary mutations and epigenetic regulation of *BRCA1/2* restore the functional BRCA proteins and contribute to PARPi resistance. Secondary mutations of *BRCA2* have been shown to restore the open reading frame and expression of functional BRCA2 proteins [62]. Of note, recent studies further demonstrate that hypomorphic *BRCA1* variants caused by genetic alterations are capable to regulate the HR activity [70], such as a *BRCA1* alternative splicing isoform without exon 11 (BRCA1-Δ11q) can induce the foci formation of RAD51 in response to DNA damage and thereby lead to PARPi resistance [70]. Promoter demethylation is another mechanism by which the BRCA1 protein can be re-expressed through transcription of epigenetically silenced *BRCA1* [71]. A preclinical study on PDX models with BRCA1-methylated ovarian cancer further showed that methylation status of all BRCA1 copies is associated with sensitivity of rucaparib [72], suggesting that complete methylation of *BRCA1* promoter might be utilized to predict the PARPi response in the clinic. Notably, the reverse mutations and epigenetic alterations associated with PARPi resistance are not exclusively detected in *BRCA1/2* but also observed in other genes involved in HR repair pathways, such as *RAD51C*, *RAD51D* and *PALB2* [73–75], providing additional biomarkers to predict the response to PARPi (Fig. 1b, upper right panel).

Several studies have demonstrated that suppression of NHEJ activity in HR-deficient tumors can restore the HR activity and regulate the PARPi resistance [76]. HR and NHEJ are the two major repair pathways for DNA double strand break repair [77]. The DNA damage response factor, 53BP1 was shown to increase the activity of NHEJ and inhibit the HR repair [78]. Previous studies showed that BRCA1 is important for removing 53BP1 from DNA ends and facilitating the transition from NHEJ to HR when DSBs happened in the S phase [79, 80]. Loss of 53BP1 restored DNA end resection and rescued the HR defects, thereby rendering BRCA1-deficient mouse mammary tumors resistant to PARPi [81]. Additionally, 53BP1 deficiency has been reported in a patient with HR restored, BRCA1-deficient breast cancer after receiving therapy of a PARPi or platinum chemotherapy [82]. Thus, loss of *BRCA1* promotes NHEJ activity and 53BP1-dependent formation of toxic chromosomal aberration in PARPi treated *BRCA1*-deficient cells, leading to hypersensitivity of PARPi [83] (Fig. 1b, lower right panel).

In addition to 53BP1 deficiency, EZH2-meditated epigenetic silencing of MAD2L2 (REV7), a critical factor involved in the 53BP1-dependent NHEJ repair pathway results in resistance to PARPi in ovarian cancer [84]. Similarly, a previous study showed that inhibiting PARylation of EZH2 promotes the EZH2-mediated epigenetic gene silencing and regulates tumor response to PARPi in *BRCA*-mutated breast cancer [85]. Moreover, loss of the end-resection antagonists, such as RIF1 [86–88] and the shieldin complex [89, 90] has been found to mediate resistance to PARP inhibitors in BRCA1-deficient tumors. PDX models with acquired resistance to PARPi were frequently associated with loss of shieldin components which comprised of SHLD1, SHLD2, SHLD3, and REV7 [90]. These end-resection antagonists were identified to block HR activity by locating at DSB sites and limiting DNA end resection [91]. Therefore, deficiency of these factors led to the recruitment of RAD51 and rescued the HR capacity in the absence of BRCA1 (Fig. 1b, lower right panel).

## Emerging strategies to overcome PARP inhibitor resistance

**Fig. 2:**
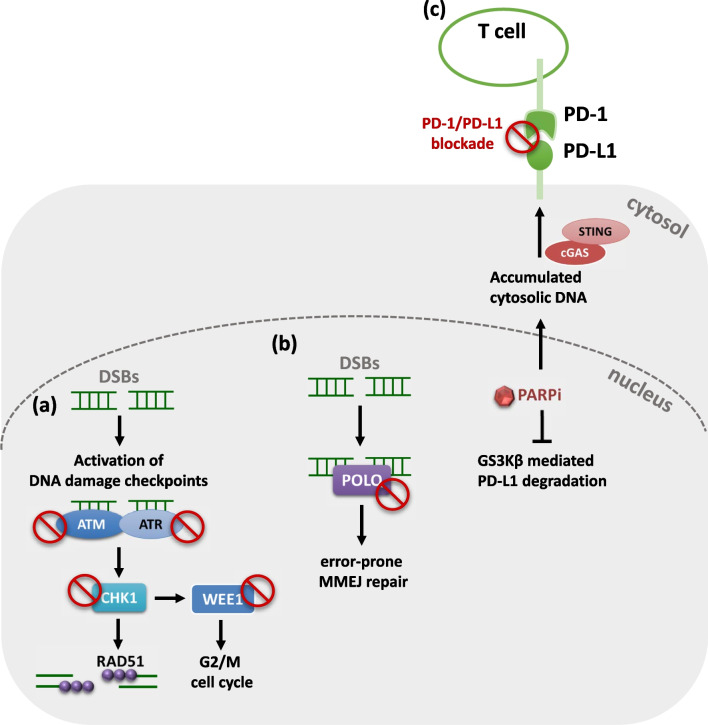
Emerging strategies to overcome PARP inhibitor resistance. **a** Inhibition of DNA damage checkpoint proteins such as ATM, ATR, CHK1 or WEE1 induces synthetic lethality with PARP inhibitor. **b** Blocking POLQ mediated MMEJ repair sensitizes tumor to PARP inhibition. **c** PARPi enhances expression of PD-L1 on cell surface by inhibiting GS3Kβ and activating cGAS-STING pathway. Combination of PD-1/PD-L1 blockade and PARPi may be the potential approaches to increase the anti-tumor activity of PARPi

Since several resistant mechanisms of PARPi have been identified, it is critical to discover the druggable targets for such mechanisms and develop the combinatorial strategies to overcome PARPi resistance. Based on the rationale of synthetic lethal interaction between PARPi and HR deficiency, therapeutic strategies that chemically induce the “BRCAness” phenotype were shown to (re)-sensitize HR-proficient or HR-restored tumors to PARPi in several cancer types. Recently, results of clinical trials evaluating PARPi in combination with inhibitors of DNA damage checkpoint proteins such as ATM, ATR, CHK1 or WEE1 demonstrated the significant efficacy of these combination by inducing synthetic lethality [92] (Fig. 2a). ATM and ATR play key roles in regulating cell cycle checkpoint signaling and induce cell cycle arrest in response to DNA damage. PARPi resistance caused by BRCA1-independent HR activity has been shown to rely on ATR-dependent RAD51 loading on DNA damage sites [93]. Of note, germline mutations of ATM compromise DSB repair and are associated with HR deficiency in patients with *BRCA*-wild type breast cancer [94]. Inhibition of another checkpoint kinase CHK1, a downstream target of ATM/ATR impairs foci formation of RAD51 and suppresses HR activity. Additionally, the G2/M checkpoint kinase, WEE1 is known to regulate G2-M cell cycle arrest to facilitate DNA repair prior to entering the mitotic phase. The combination of PARPi and WEE1 inhibitors has exhibited the significant synergistic effects in preclinical models [95], and currently, there are many ongoing clinical trials evaluating this combination in patients with different cancer types [92]. Furthermore, a recent study identified a novel druggable target, DNA polymerase theta (POLQ), which is highly expressed in HR-deficient ovarian and breast tumor [96]. POLQ has been shown to regulate DSB repair by the error-prone microhomology-mediated end-joining (MMEJ) pathway to compensate the impaired HR activity in HR-deficient tumors [96, 97]. Notably, preclinical studies demonstrated the synthetic lethal interaction between POLQ inhibitors and PARPi in HR-deficient tumors with acquired resistance to PARPi [98, 99] (Fig. 2b). The POLQ inhibitor, ART4215 recently entered the phase I/II clinical trial in combination with talazoparib for the treatment of patients with metastatic breast cancer. These findings suggest that POLQ inhibitors hold great potential to overcome the acquired resistance to PARPi in HR-deficient tumors. Although the pre-clinical and clinical studies combining DDR inhibitors with PARPi demonstrated significant anti-tumor effects [100], targeting multiple proteins in the DNA damage response pathways is frequently limited by overlapping toxicities to non-malignant cells [101].

Due to the discovery of PARPi in regulating immune responses, combination of immune checkpoint inhibitors and PARPi may be the potential approaches to increase the anti-tumor activity of PARPi. In particular, PARPi is shown to enhance the PD-L1 expression and immunosuppressive effects via inhibition of GS3Kβ-mediated PD-L1 degradation [102]. Similarly, PARP inhibition induces the cytosolic accumulation of DNA fragments and activates the cGAS-STING signaling pathway to increase the surface expression of PD-L1 [103]. Furthermore, these studies also demonstrated that PARPi increases CD8^+^ T cell infiltration in tumors and promotes the anti-tumor effects of the PD-1/PD-L1 blockade in mouse models [102, 103] (Fig. 2c). Several clinical trials are currently underway investigating the anti-tumor effects of immune checkpoint inhibitors in combination with PARPi in several cancer types [104, 105]. Although results from a clinical trial demonstrated that the combination of niraparib and pembrolizumab is well tolerated and associated with promising signals of activity [104], further clinical studies are needed to validate these findings.

## Marker-guided effective therapy (Mget) strategies to overcome resistance to PARPi

**Fig. 3:**
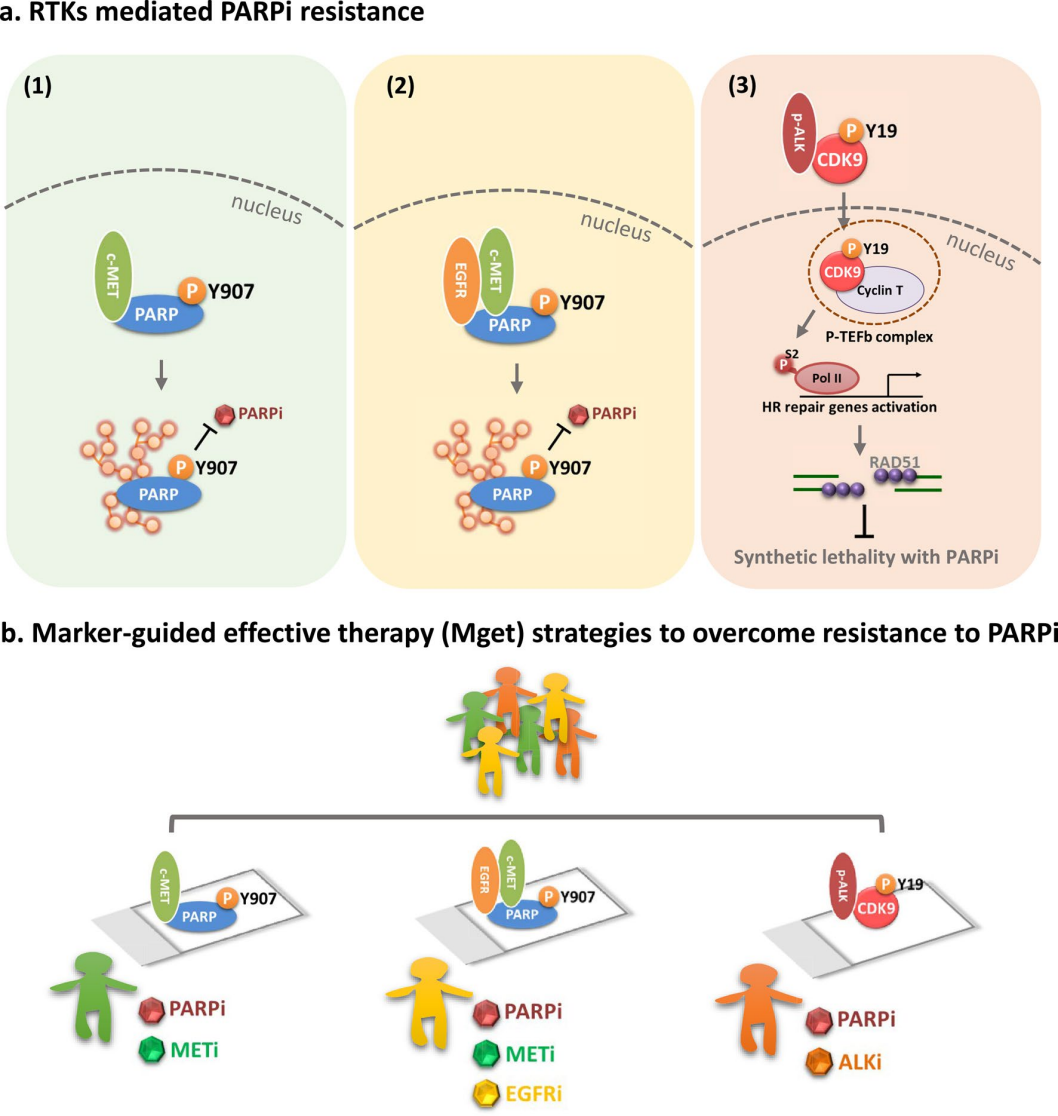
Overcoming resistance to PARPi by Marker-guided effective therapy (Mget) strategies. **a** RTK-mediated PARPi resistance. (1) c-MET phosphorylates PARP1 at Y907 to upregulate PARylation and prevent the binding of PARPi, thereby resulting in PARPi resistance. (2) Heterodimerization of EGFR and c-MET contributes to PARPi resistance through phosphorylation of PARP1-Y907. (3) Phosphorylated ALK (p-ALK) promotes HR activity and PARPi resistance by directly phosphorylating CDK9 at Y19. p-Y19 CDK9 facilitates nuclear localization of CDK9 and interacts with cyclin T to form a p-TEFb (CDK9/cyclin T) complex, which turns on transcription of HR repair genes. **b** Marker-guided effective therapy (Mget) strategies to overcome resistance to PARPi. Expression of RTKs and their specific phosphorylated substrates detected in patient tumor tissues could be utilized to predict PARPi resistance and select the right patients for treatment with PARPi in combination with the specific RTK inhibitors

Although there are an increasing number of clinical trials evaluating PARPi in combination with other agents in several different cancer types [31], the lack of predictive biomarkers for guiding the combination therapy may limit their efficacy because responders and non-responders cannot be discriminated. Targeting oncogenic protein kinases have been shown to sensitize tumors to PARPi through regulating enzyme activity of PARP1 or indirectly inhibiting the HR machinery. Several studies reported that VEGFR [106], EGFR [107], or IGF1R [108] contribute to PARPi resistance through restoring the HR activity. Notably, recent studies further identified some of RTKs mediated phosphorylation of their downstream substrates could be utilized as biomarkers to predict the resistance to PARPi and guide rational combination of PARP and RTK inhibitors (Fig. 3). Specifically, c-MET is shown to directly interact with PARP1 and phosphorylate it at Y907 residue. The phosphorylation of Y907-PARP1 (p-Y907 PARP1) upregulates the enzymatic activity of PARP1 and prevents the binding of PARPi, thereby resulting in resistance to PARPi [58]. Importantly, expression of p-Y907 PARP1 is positively associated with expression of c-MET in the tumor tissues of breast cancer patients, and combination of c-MET and PARPi synergistically suppresses the growth of xenograft tumors which have high c-MET and p-Y907 PARP expression. In addition to breast cancer, c-MET/p-Y907 PARP axis meditated PARPi resistance has also been demonstrated in other cancer types, including ovarian [109] and pancreatic cancer [110]. Additionally, the abundant expression of p-Y907 PARP was also identified in the tumor tissues of patients with hepatocellular carcinoma (HCC) [97]. Interestingly, EGFR was found to interact with c-MET and phosphorylate PARP-Y907 in the HCC cells that have high EGFR and c-MET expression, and simultaneous inhibition of both EGFR and c-MET significantly increases the anti-tumor activity of PARPi in such HCC cells [111]. This finding has also been identified in the TNBC cells with acquired resistance to PARPi, suggesting that heterodimerization of EGFR and c-MET plays key role in PARPi resistance [112]. Most recently, another receptor tyrosine kinase, ALK was shown to promote HR activity and PARPi/platinum resistance through phosphorylating CDK9 at Y19 residue (p-Y19 CDK9) in ovarian and breast cancer [113]. Mechanistically, the phosphorylated ALK (p-ALK)/p-Y19 CDK9 kinase cascade stabilizes positive transcription elongation b complex (P-TEFb), and in turn, activates RNA Pol II-dependent transcription of genes involved in the HR pathway, resulting in PARPi resistance (Fig. 3a). Notably, combination of FDA-approved ALK and PARP inhibitors significantly suppressed tumor growth and prolonged animal survival in PARPi/platinum-resistant tumor xenograft models. Importantly, p-ALK expression is associated with resistance to PARPi and positively correlated with p-Y19-CDK9 expression in the human tumor tissues. This study provided the preclinical and clinical data in support of a marker-guided, PARPi-based combinatorial effective therapy which leverages synthetic lethality by targeting ALK [113]. Collectively, these findings suggest that expression of RTKs and their specific phosphorylated substrates (e.g. c-MET/p-Y907 PARP, EGFR/c-MET/p-Y907 PARP and p-ALK/p-Y19 CDK9) can be utilized to select patients whose tumors have a high likelihood of responding to combined inhibition of PARP and RTK (Fig. 3b). Furthermore, because these RTK inhibitors are currently used in the clinic, these promising combinatorial treatment strategies involving RTK and PARPi are expected to be rapidly translated into clinic.

## Conclusions and future perspective

In conclusion, the promising effects of PARPi in several cancer types have been highlighted by an increasing number of preclinical and clinical studies, showing their therapeutic benefits over conventional chemotherapy in a substantial population of patients. Moreover, knowledge of molecular mechanisms driving the efficacy of and resistance to PARPi has led to the development of multiple PARPi-based combination strategies. However, how to select the right patients for treatment with PARPi either as monotherapy or in combination with other agents remains an unmet need in the clinic. Therefore, further detailed mechanistic studies of PARPi resistance, along with pre- and post- treated patient samples from clinical trials will help us to maximize the use of PARPi in the clinic. Moreover, it is necessary to identify more reliable biomarkers for selecting appropriate patients, which may be identified by multi-omics strategies in patient samples with the corresponding clinical data.

## Data Availability

Not applicable.

## Abbreviations

ALK: Anaplastic lymphoma kinase
BER: Base excision repair
c-MET: Mesenchymal-epithelial transition factor
DSBs: Double-strand breaks
EGFR: Epidermal growth factor receptor
HCC: Hepatocellular carcinoma
HR: Homologous recombination
NHEJ: Non-homologous end joining
PARP: Poly (ADP-ribose) polymerase (PARP)
PARylation: Poly(ADP-ribosyl)ation
P-TEFb: Positive transcription elongation b complex
PD-L1: Programmed death-ligand 1
POLQ: DNA polymerase theta
RTK: Receptor tyrosine kinase
TNBC: Triple negative breast cancer
Tyr or Y: Tyrosine
